# Effects of different bio-organic fertilizers on agronomic traits, yield, and dry matter accumulation of fresh-eating corn in arid and saline soils of Xinjiang: a single-site field experiment

**DOI:** 10.3389/fpls.2026.1866274

**Published:** 2026-06-05

**Authors:** Yanhua Cui, Yong Tang, Yao Du, Wanhong Ding, Hongsong Ren, Yulong Jia, Xuming Shi, Wei Wang, Shun Ji, Yibo Ma

**Affiliations:** 1 Xinjiang Uygur Autonomous Region Leisure Agriculture Engineering Research Center, Xinjiang Academy of Agricultural Sciences, Urumqi, China; 2 Urumqi Comprehensive Experiment Station, Xinjiang Academy of Agricultural Sciences, Urumqi, China; 3 College of Computer Science and Information Engineering, Anyang Institute of Technology, Anyang, China

**Keywords:** agronomic traits, bio-organic fertilizer, dry matter accumulation, fresh-eating corn, yield

## Abstract

Fresh-eating corn is an important economic crop in Xinjiang, while its yield and quality are severely limited by drought, soil salinization and low soil fertility. Applying bio-organic fertilizers can effectively mitigate abiotic stress and improve crop growth performance, yet suitable fertilizer types for the main cultivar Wannuo 2000 remain unclear in arid and salinized regions. A two-year consecutive field experiment was conducted from 2024 to 2025. Combined with field meteorological data, short-term low sunlight stress in late June 2024 significantly affected crop growth and interannual growth stability. On the basis of growth indices and yield traits, two kinds of membership function evaluation methods were adopted to comprehensively assess fertilizer application effects. The results showed that different functional bio-organic fertilizers had distinct regulatory effects on agronomic traits, ear development, dry matter accumulation and yield formation. Among all treatments, Tianwu Jintaiyang bio-organic fertilizer exhibited the best comprehensive performance with high yield and strong environmental adaptability. Correlation analysis indicated that ear grain weight, husked ear weight and kernel number per row were key positive factors for yield improvement, whereas bald tip length and barren stalk rate were the main limiting factors. This study clarifies the differentiated regulation effects and regional adaptability of various bio-organic fertilizers, and provides theoretical basis and technical guidance for rational fertilization and green stable high-yield cultivation of fresh-eating corn in arid salinized farmlands of Xinjiang.

## Introduction

Food security and green agricultural development are major concerns worldwide. With increasing soil degradation, drought stress, and salinization, reducing chemical fertilizer input and improving farmland ecosystem stability have become critical for sustainable crop production (Zhang et al., 2015; Zhao et al., 2022; Wang, 2025). Fresh-eating corn is an economically important specialty crop in China, and its industrial development is of great significance for ensuring farmers’ income and food structure diversity (Aliyu et al., 2022; Bao et al., 2024; Shim et al., 2026). As a typical high-quality waxy fresh corn variety, *Wannuo 2000* has early maturity, superior taste, high amylopectin content, and wide adaptability. It has become the dominant cultivar in Huang-Huai-Hai and Northwest China (Luo et al., 2020; Li et al., 2022; Liu et al., 2022). In Xinjiang, the planting proportion of *Wannuo 2000* accounts for more than 35% of the total fresh-eating corn area, supporting the development of characteristic high-efficiency agriculture (Amuti et al., 2018; Zhu et al., 2024; Zhang et al., 2025b).

Xinjiang possesses abundant light and heat resources, large diurnal temperature difference, and favorable conditions for corn quality improvement. However, the sustainable production of fresh-eating corn is severely restricted by multiple abiotic stresses in this region (He et al., 2023; Chen et al., 2024; Qu et al., 2024). The soil organic matter content of local irrigated desert soil is generally lower than 1.0%, with poor nutrient retention capacity (Ning et al., 2023a; Cui et al., 2025). Meanwhile, seasonal drought is prominent, with annual precipitation during the growth period less than 50 mm. Additionally, soil secondary salinization is common, and the electrical conductivity of topsoil can reach 1.80–2.50 mS/cm. These constraints jointly inhibit root growth, nutrient absorption, and ear development, often leading to increased bare tip length, higher empty-stalk rate, and unstable yield (Ning et al., 2023b; Sun et al., 2025). Long-term over-application of nitrogen fertilizer further aggravates soil compaction and quality degradation, which seriously limits the yield and commercial performance of *Wannuo 2000* (Zhou et al., 2021, 2022). Therefore, developing green and efficient fertilization technologies suitable for arid and salinized environments is urgently needed to break production bottlenecks.

Bio-organic fertilizers combined with functional microbial strains can improve soil structure, enhance water and fertilizer retention, alleviate salt and drought stress, and promote crop growth. Recent studies confirmed that bio-organic fertilizers effectively improve rhizosphere microenvironment, enhance stress resistance, and optimize ear development in maize (Zhang et al., 2025a; Zhao et al., 2025). In arid and salinized regions, bio-organic fertilizers have become an important technical approach to improve soil quality and crop productivity (Zhang et al., 2015; Zhao et al., 2018; Zhang et al., 2026). By supplying organic materials and functional bacteria, these fertilizers can synchronize nutrient release with crop demand, delay leaf senescence, promote grain filling, and reduce the occurrence of bare tip and empty-stalk (Ning et al., 2023a; Dang and Chuong, 2026). However, most studies focus on normal soil conditions, and there is still a lack of systematic screening and evaluation of specialized bio-organic fertilizers for Wannuo 2000 under arid and salinized soil conditions in Xinjiang. The regulatory mechanism of different functional bio-organic fertilizers on agronomic traits, dry matter accumulation, and yield stability remains unclear.

Accordingly, this study conducted a two-year fixed-point field experiment to explore the effects of six bio-organic fertilizers on agronomic traits, ear characteristics, yield, and dry matter accumulation of fresh-eating corn Wannuo 2000. We hypothesize that bio-organic fertilizers containing salt-tolerant and drought-tolerant functional strains can effectively improve root microenvironment, alleviate abiotic stress, optimize ear development, reduce bare tip length and empty-stalk rate, and thereby enhance yield and stability. The objectives were to screen the most suitable bio-organic fertilizer, clarify the key factors affecting yield, and provide a theoretical basis and technical support for green, high-yield, and high-quality cultivation of fresh-eating corn in arid and salinized regions of Xinjiang.

## Materials and methods

### General situation of the experimental field

**Figure 1:**
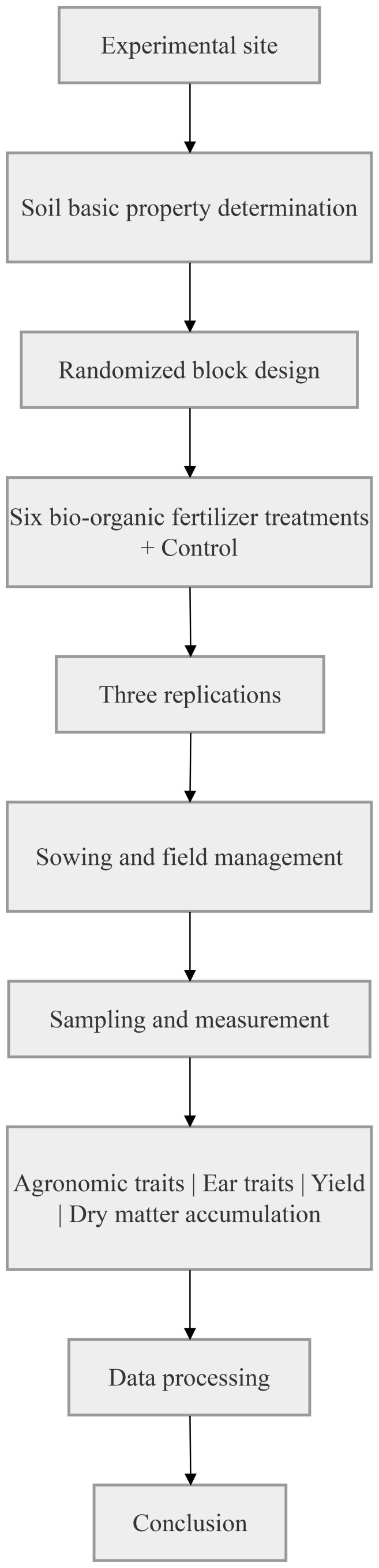
Schematic diagram of the experimental technical route.

This experiment was a two-year consecutive field fixed - point experiment carried out from 2024 to 2025 at the Urumqi Comprehensive Experimental Station of Xinjiang Academy of Agricultural Sciences. The experimental site is located at 43°56′48″ N, 87°28′32″ E, with an altitude of 522 meters. The site has a typical temperate continental arid climate, with an average annual temperature of 7.5–8.0 °C and annual precipitation less than 200 mm, of which less than 50 mm falls during the corn growing season. The key meteorological elements during the fresh corn growing season differed slightly between the two years. The accumulated temperature was 2428.4 °C in 2024 and 2415.4 °C in 2025, the average diurnal temperature difference was 11.8 °C and 11.9 °C, and the total growing-season precipitation was 87.5 mm and 87.8 mm, respectively. Although the overall hydrothermal conditions were similar, there existed obvious light condition differences in critical growth stages. Specifically, five consecutive days with daily sunshine duration lower than 5 hours occurred in late June 2024, which coincided with the ear differentiation stage of fresh corn, while no such low-sunlight adverse weather appeared in 2025. The soil at the experimental site (0–20 cm layer) is an irrigated desert soil according to the Chinese Soil Classification System, with a pH of 8.46, electrical conductivity (EC) of 2.18 mS·cm^-1^, bulk density of 1.42 g·cm^-3^, organic matter content of 11.32 g·kg^-1^, total nitrogen of 0.68 g·kg^-1^, nitrate nitrogen of 8.25 mg·kg^-1^, available phosphorus of 12.45 mg·kg^-1^, and available potassium of 168.32 mg·kg^-1^. The previous crop was wheat, and field management practices were kept consistent across all treatments. **Figure 1** shows the schematic diagram of the experimental process, including experimental design, field management, sampling, measurement, and data analysis.

### Test crops

The test crop is the fresh-eating corn variety Wannuo 2000, with the approval number of Guoshenyu 2016008. It was bred by Hebei Huasui Seed Industry Co., Ltd. This variety belongs to the medium-early-maturing white-waxy type, with a whole growth period of 80–85 days. It is a dominant variety in the main production areas of Xinjiang, and its planting area accounted for more than 35% in 2024. Wannuo 2000 has a semi-compact plant type. Its plant height is 220–240 cm, the ear height is 80–90 cm, and the stem diameter is 2.30-2.50cm, showing relatively strong lodging resistance. The ear is cylindrical, 20.00-22.00 cm long, with 14–16 rows per ear, and the commercial ear rate is not less than 90%. In terms of quality, the amylopectin content of the grains is not less than 98%, and the soluble sugar content is 12.00%-15.00%. After cooking, it tastes soft, glutinous and fragrant. Regarding stress resistance, it is highly resistant to northern corn leaf blight and stalk rot, moderately resistant to southern corn leaf blight, and can adapt to the arid and high-temperature climate in Xinjiang. However, it is relatively sensitive to soil salinization. When the electrical conductivity of the plow layer is higher than 2.5 mS/cm, the empty-stalk rate increases significantly.

### Test fertilizers

A total of 7 treatments were set up in the experiment, including 6 bio-organic fertilizer/microbial fertilizer treatments and 1 blank control. The specific information is shown in **Table 1**.

**Table 1:** Detailed information of the tested bio-organic fertilizers / microbial inoculants.

Treatment	Fertilizer name	Manufacturer	Core functional components	Technical specifications	Application rate
T1	Lüzhou Dayang Bio-organic Fertilizer	Xinjiang Lüzhou Dayang Biotechnology Co., Ltd.	Salt-tolerant strain (Bacillus licheniformis), Humic acid	Viable bacterial count ≥ 2.0×10^8^ cfu/g, Organic matter ≥ 45%, Salinization amelioration rate ≥ 15%	2250 kg/ha
T2	Composite Microbial Fertilizer Lüzhou Dayang	Xinjiang Lüzhou Dayang Biotechnology Co., Ltd.	Nitrogen-fixing bacteria (Azotobacter chroococcum), Phosphate-solubilizing bacteria (Bacillus megaterium)	Viable bacterial count ≥ 5.0×10^8^ cfu/g, N+P_2_O_5_+K_2_O ≥ 6%	1800 kg/ha
T3	Dayitian Bio-organic Fertilizer	Shandong Dayitian Fertilizer Industry Co., Ltd.	Compound probiotic consortium, Amino acids	Viable bacterial count ≥ 2.0×10^8^ cfu/g, Organic matter ≥ 40%, Amino acids ≥ 5%	2250 kg/ha
T4	Earthworm Enzyme Bacteria Bio-organic Fertilizer	Henan Earthworm Enzyme Biotechnology Co., Ltd.	Earthworm protease, Potassium-solubilizing bacteria (Bacillus mucilaginosus)	Viable bacterial count ≥ 2.0×10^8^ cfu/g, Organic matter ≥ 42%, Protease activity ≥ 150 U/g	2025 kg/ha
T5	Tianwu Jinyang Bio-organic Fertilizer	Shanxi Tianwu Jinyang Fertilizer Industry Co., Ltd.	Drought-resistant strain (Halomonas campaniensis), Humic acid	Viable bacterial count ≥ 2.0×10^8^ cfu/g, Organic matter ≥ 45%, Water retention rate ≥ 8%	2250 kg/ha
T6	Keshike Li Microbial Inoculant	Beijing Keshike Li Biotechnology Co., Ltd.	High-efficiency nitrogen-fixing bacteria (Rhizobium sp.), Biostimulants	Viable bacterial count ≥ 1.0×10^9^ cfu/g, Biostimulants ≥ 3%	900 kg/ha
T7	CK (Blank Control)	–	–	–	0

### Experimental design

This experiment employed a randomized complete block design (RCBD) with three replications. Randomization was performed using a random number table to independently assign each treatment to the corresponding plot, ensuring uniform spatial distribution and unbiased arrangement. Each plot had an area of 40 m². The planting specifications consisted of a plant spacing of 30 cm and a row spacing of 60 cm, resulting in a planting density of approximately 55,556 plants/hm². To eliminate edge effects and prevent interference from lateral fertilizer seepage between different treatments, a 0.50 m wide isolation protection row was established between adjacent plots. The experiment was conducted continuously in 2024 and 2025. The sowing date was May 14th in both years, and the harvest date was in late August. To minimize potential observer bias, single-blinding was implemented during the measurement of agronomic traits, ear morphology, and yield components; investigators were not informed of the corresponding treatment group during data collection. All field management measures throughout the entire growth period remained consistent across treatments.

### Field management measures

Standardized field management was carried out throughout the experiment. The direct-seeding method was used for sowing, with 2 seeds sown per hole. After emergence, seedlings were promptly thinned, and 1 robust seedling was left per hole. All bio-organic fertilizers were applied as base fertilizers. They were evenly spread and plowed into the soil one week before sowing, in conjunction with land preparation, to ensure a uniform distribution of nutrients. Irrigation adhered to the principle of supplying water as needed. Water was supplemented in a timely fashion according to soil moisture conditions, with an emphasis on ensuring water supply during key growth stages such as the jointing and silking stages. Regarding pest and disease control, green prevention and control standards were strictly adhered to. Aphids were trapped by hanging yellow boards, and Trichogramma was released to control corn borers. Chemical pesticides were prohibited during the entire growth period. Weed control adopted a combination of chemical closure and manual assistance. Specifically, pre-emergence herbicides were sprayed for soil closure treatment after sowing but before emergence, and manual weeding was carried out in combination with inter-cultivation and soil-banking during the seedling stage to ensure that there was no weed infestation in the field.

### Sample collection and determination

During the harvest period, 10 representative plants were selected from each plot to measure their agronomic traits. These traits included plant height, ear height, the ratio of ear height to plant height, stem diameter, ear length, ear diameter, bare tip length, the number of ear rows, the number of grains per row, cob diameter, etc. The empty-stalk rate, lodging rate, and stalk-breakage rate were also recorded. At the same time, the grain weight per ear, the weight of an ear without husk, the weight of an ear with husk, 100-grain weight, and cob weight were measured. The plot yield was determined by harvesting and weighing all fresh ears in the plot. All investigation and variety-evaluation methods were conducted in accordance with “Description Specifications and Data Standards for Maize Germplasm Resources” (GB/T 3543.1 - 1995).

### Data analysis

Data entry and preliminary organization were performed using Microsoft Excel 2021 (Microsoft Corporation, Redmond, WA, USA). Data cleaning was conducted using Pandas (version 1.5.3), and charts were generated using Matplotlib (version 3.7.1) within the Python environment. Statistical analyses, including variance and correlation analyses, were carried out using SPSS 26.0 (IBM Corp., Armonk, NY, USA). The Pearson correlation coefficient was calculated to analyze the correlations among various indicators. Two evaluation approaches including equal-weight membership function and entropy weight membership function were adopted for multi-index comprehensive evaluation. The equal-weight comprehensive value was obtained by simple average of all single-index membership values (Tian et al., 2024). The entropy weight method was applied to calculate objective weight of each index, and then weighted comprehensive membership value was further determined (Yu et al., 2019). Combined application of the two methods could improve the rationality and credibility of evaluation results.

## Results

### Effects of different fertilization treatments on agronomic traits of fresh-eating corn

#### Plant height and ear height

**Figure 2:**
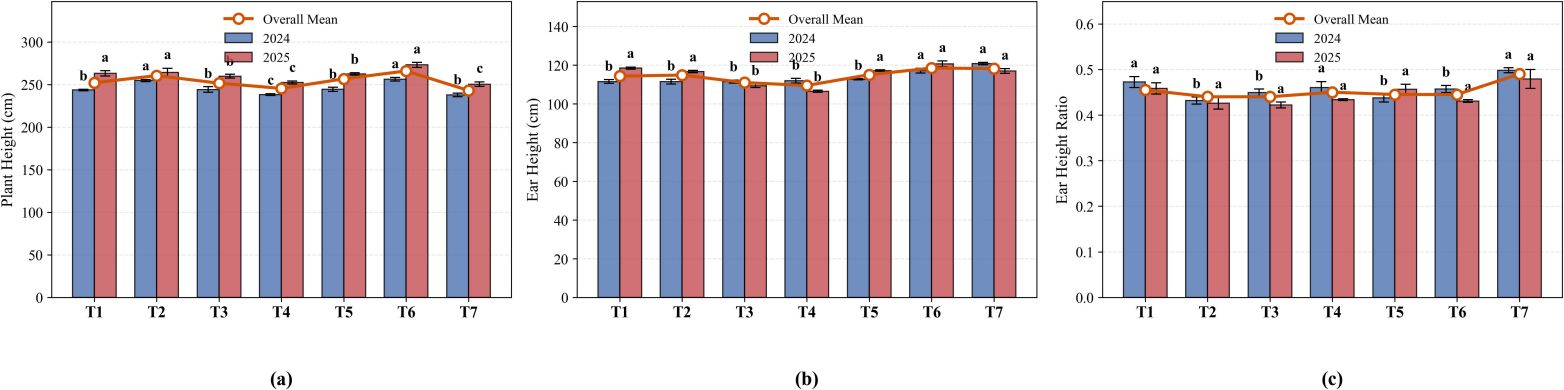
Effects of different fertilization treatments on main agronomic traits of fresh-eating corn **(a)** Plant height; **(b)** Ear height; **(c)** Ratio of ear height to plant height. Error bars represent standard deviation; different lowercase letters indicate significant differences at *P* < 0.05 level.

Different fertilization treatments had a significant impact on the plant height of fresh-eating corn. As shown in **Figure 2**, the average plant height over the two years was in the following order: T6 (265.83 ± 7.83cm) > T2 (260.38 ± 7.28 cm) > T5 (256.54 ± 7.56 cm) > T1 (251.99 ± 6.79 cm) > T3 (251.50 ± 7.34 cm) > T4 (245.31 ± 7.22 cm) > T7 (242.95 ± 7.11 cm). Among these, treatment T6 had the highest plant height, which was significantly increased by 9.4% compared to the CK. Treatments T2 and T5 came next, with increases of 7.20% and 5.60% respectively compared to the CK. The trend of ear height change was basically the same as that of plant height. Treatment T6 had the highest ear height (118.60 ± 4.41 cm), followed by treatment T5 (116.80 ± 4.37 cm), while the CK had the lowest (110.20 ± 4.32 cm). The ratio of ear height to plant height was the highest in treatment T4 (0.466 ± 0.014) and the lowest in treatment T6 (0.446 ± 0.015), indicating that the plants in treatment T6 had a lower center of gravity and stronger lodging resistance.

#### Stem diameter and stress resistance traits

**Figure 3:**
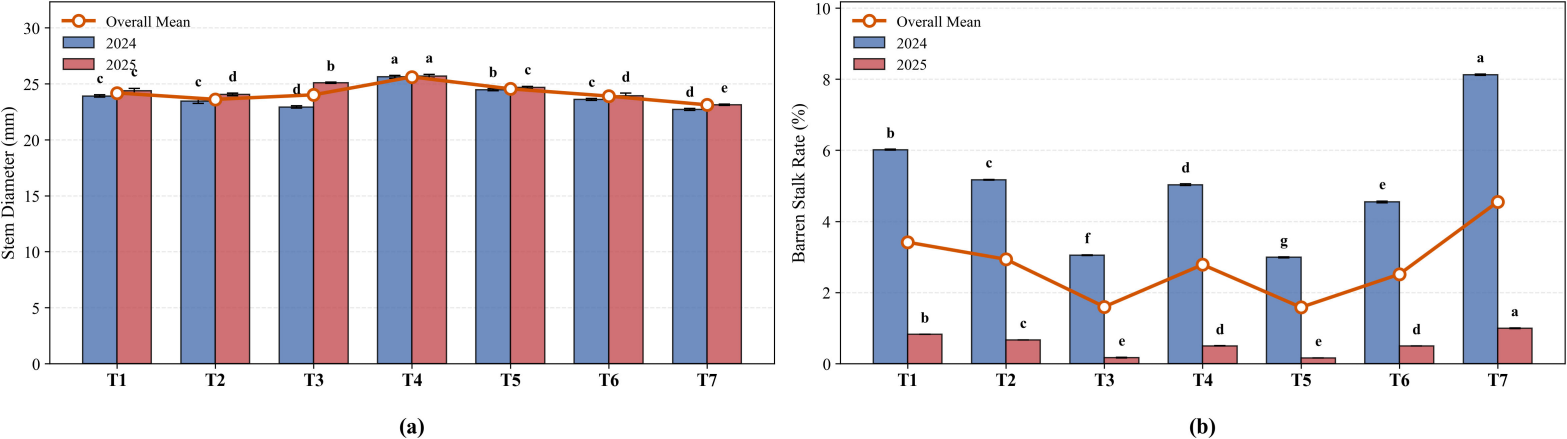
Effects of different fertilization treatments on main agronomic traits of fresh-eating corn **(a)** Stem diameter; **(b)** Barren stalk rate. Error bars represent standard deviation; different lowercase letters indicate significant differences at *P* < 0.05 level.

The T4 treatment had the largest stem diameter (25.61 ± 1.01mm), which was significantly greater than that of other treatments, representing a 10.8% increase compared to the CK. Next were the T5 (24.54 ± 0.91mm) and T1 (24.18 ± 0.87 mm) treatments, while the CK treatment had the smallest stem diameter (23.11 ± 0.76 mm) (**Figure 3**). Stem diameter is a vital indicator for assessing a plant’s lodging resistance; the largest stem diameter in the T4 treatment implies it has the greatest potential for lodging resistance. During the experimental period, the lodging rate and stalk - breaking rate were 0% for all treatments. This result is due to stable climatic conditions (lack of extreme wind and heavy rain) and effective field management. The barren - stalk rate was in the order: T7 (4.55 ± 1.10%) > T1 (3.42 ± 0.82%) > T4 (2.79 ± 0.67%) > T2 (2.94 ± 0.71%) > T6 (2.52 ± 0.60%) > T3 (1.60 ± 0.41%) > T5 (1.58 ± 0.40%). The T5 treatment had the lowest barren - stalk rate, indicating the best uniformity in population growth.

### Effects of different fertilization treatments on ear traits of fresh-eating corn

#### Ear length, ear diameter, and bald tip length

**Figure 4:**
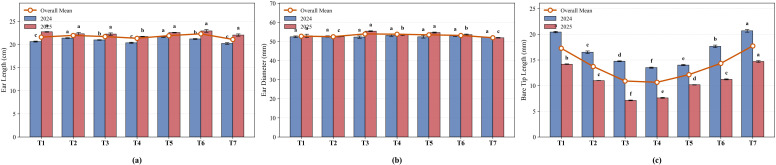
Effects of different fertilization treatments on main ear traits of fresh-eating corn **(a)** Ear length; **(b)** Ear diameter; **(c)** Bald tip length. Error bars represent standard deviation; different lowercase letters indicate significant differences at *P* < 0.05 level.

Ear traits are key factors influencing the yield and commercial quality of fresh-eating corn. As shown in **Figure 4a**, different fertilization treatments had significant effects on ear length. The two-year average ear length followed the order: T6 (22.30 ± 1.13 cm) > T2 (21.95 ± 0.77 cm) > T5 (21.90 ± 0.74 cm) > T3 (21.71 ± 0.74cm) > T1 (21.60 ± 1.04 cm) > T4 (21.33 ± 1.12 cm) > T7 (21.09 ± 1.39cm). The T6 treatment produced the longest ears, representing a 5.70% increase compared to the CK, followed by the T2 and T5 treatments, which showed increases of 4.10%-4.30% relative to the CK. Ear diameter (**Figure 4b**) followed the order: T3 (53.95 ± 1.37 mm) > T4 (53.78 ± 0.96 mm) > T5 (53.46 ± 0.95 mm) > T6 (53.16 ± 0.73 mm) > T1 (52.77 ± 0.14 mm) > T2 (52.50 ± 0.25 mm) > T7 (51.95 ± 0.09 mm). All fertilized treatments exhibited greater ear diameters than the CK, with increases ranging from 1.10% to 3.80%. Bald tip length is an important indicator of commercial quality in fresh-eating corn; shorter bald tips correspond to better marketability. As shown in **Figure 4c**, T4 treatment obtained the minimum bald tip length of 10.64 ± 4.16 mm, which was 34.20% lower than that of the control group. T5 and T3 treatments also showed obvious effects in reducing bald tip length, with corresponding values of 12.13 ± 2.87 mm and 10.89 ± 5.36 mm, respectively. By contrast, T1 and CK treatments presented relatively longer bald tip length, both exceeding 17 mm. The above findings demonstrated that the application of earthworm enzyme-based bio-organic fertilizer and Tianwu Jintaiyang bio-organic fertilizer could effectively shorten bald tip length and improve the commercial ear quality of fresh-eating corn.

#### Kernel row number, kernel number per row, and cob traits

**Figure 5:**
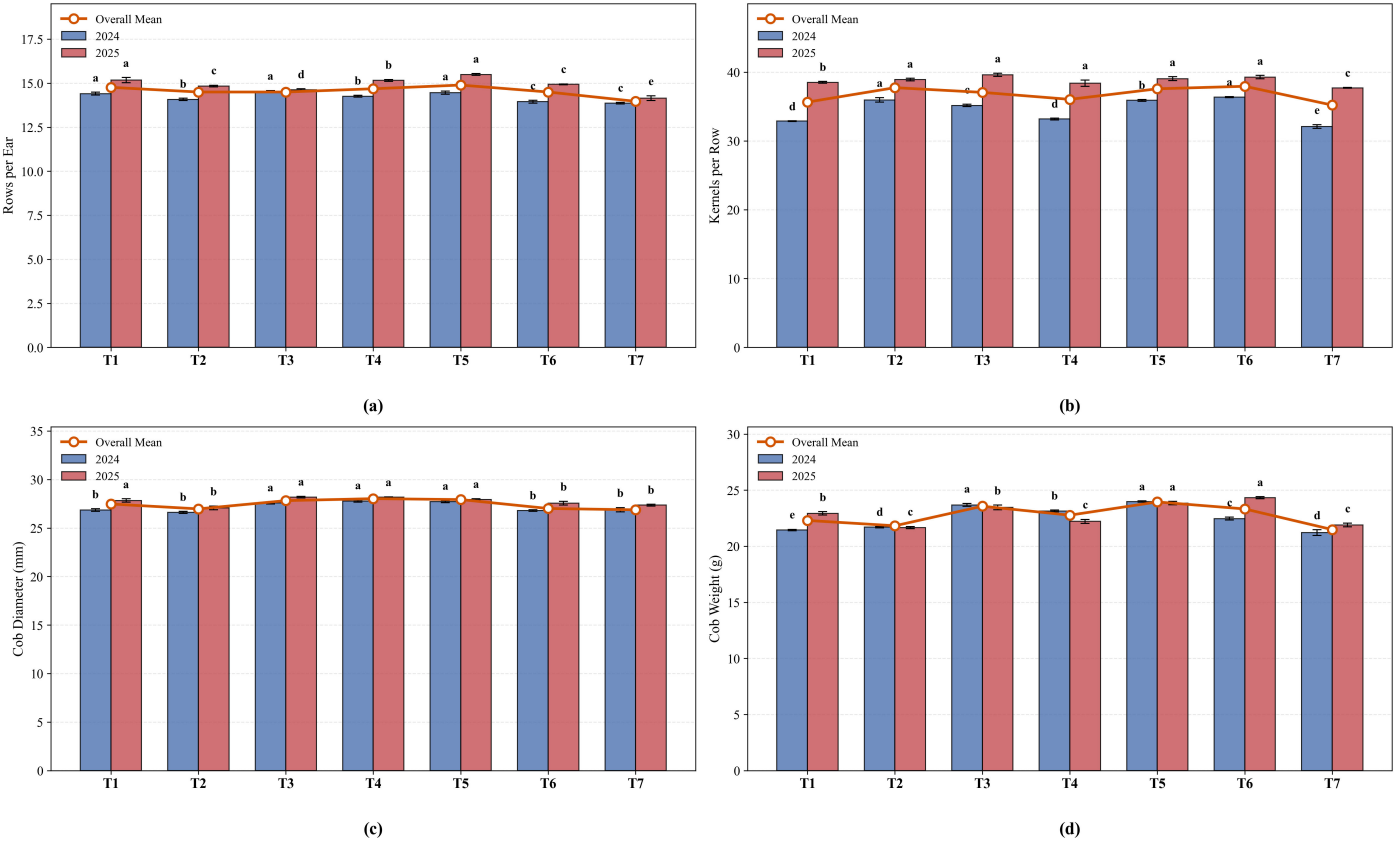
Effects of different fertilization treatments on main ear and kernel traits of fresh-eating corn **(a)** Kernel row number; **(b)** Kernel number per row; **(c)** Cob diameter; **(d)** Cob weight. Error bars represent standard deviation; different lowercase letters indicate significant differences at *P*<0.05 level.

As shown in **Figure 5**, the number of kernel rows per ear did not differ significantly among treatments, ranging from 14 to 16 rows. The number of kernels per row was highest in the T6 treatment (37.94 ± 1.89 kernels), followed by the T2 treatment (37.73 ± 1.89 kernels), while the CK treatment had the fewest kernels (35.20 ± 4.29 kernels). Cob diameter followed the order: T4 (28.02 ± 0.06 mm) > T5 (27.93 ± 0.07 mm) > T3 (27.82 ± 0.41 mm) > T1 (27.48 ± 0.69 mm) > T6 (27.01 ± 0.57mm) > T2 (26.91 ± 0.41 mm) > T7 (26.88 ± 0.50 mm). Cob weight was highest in the T5 treatment (23.95 ± 0.11 g), followed by the T6 treatment (23.32 ± 1.21 g), with the CK treatment showing the lowest value (21.48 ± 0.26 g).

### Effects of different fertilization treatments on yield and related traits of fresh - eating corn

#### Components of single - plant yield

**Figure 6:**
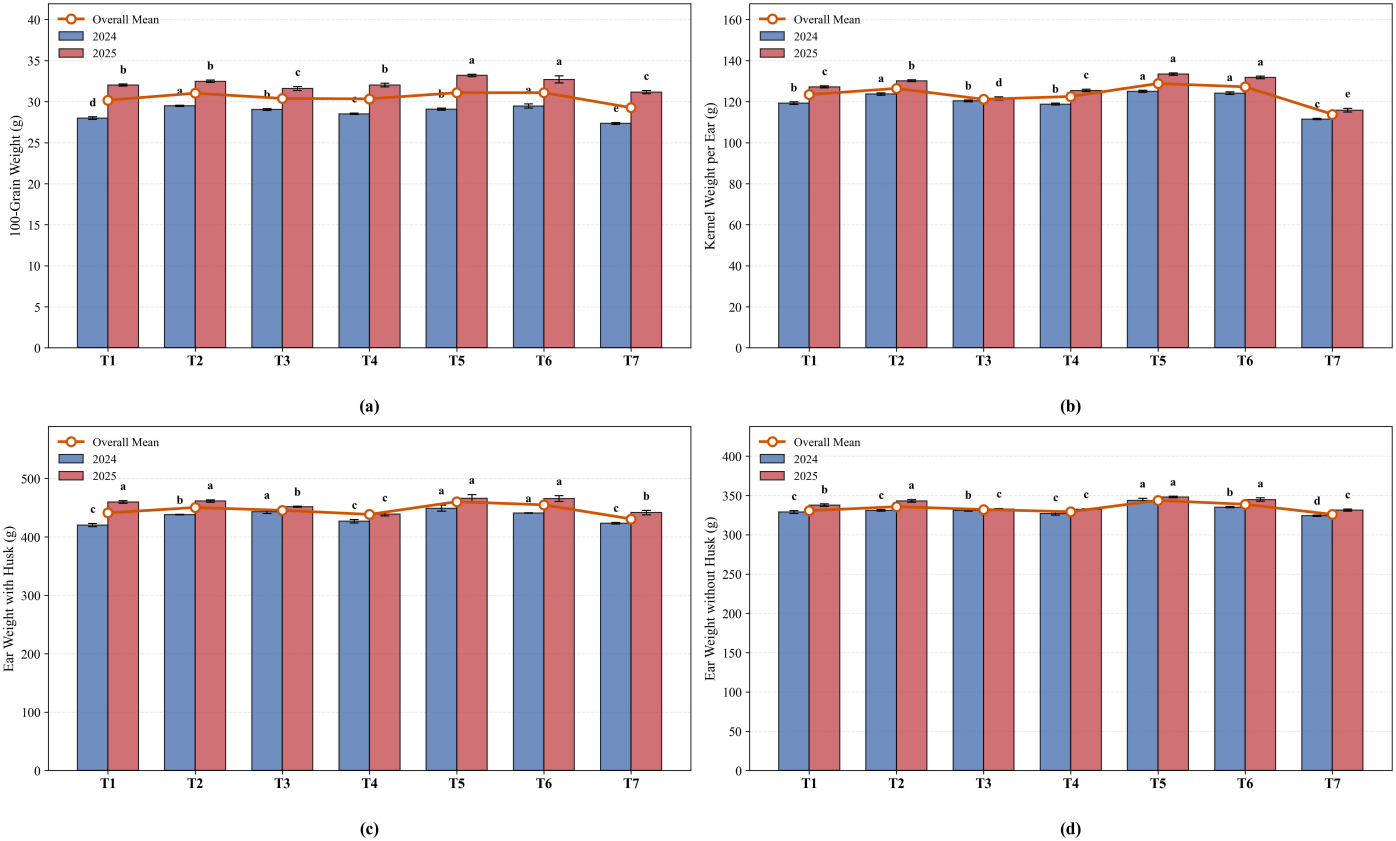
Effects of different fertilization treatments on yield-related traits of fresh-eating corn **(a)** 100-kernel weight; **(b)** Kernel weight per ear; **(c)** Fresh ear weight with husk; **(d)** Fresh ear weight without husk. Error bars represent standard deviation; different lowercase letters indicate significant differences at *P*<0.05 level.

As depicted in **Figure 6**, different fertilization treatments had significant impacts on the yield traits of fresh - eating corn. The two - year average kernel weight per ear was in the order: T5 (128.82 ± 6.54 g) > T6 (127.20 ± 6.34 g) > T2 (126.43 ± 5.95 g) > T1 (123.43 ± 7.67 g) > T4 (122.49 ± 4.41 g) > T3 (121.13 ± 0.90 g) > T7 (113.82 ± 3.51 g). The T5 treatment had the highest kernel weight per ear, showing a 13.2% increase compared to the CK, with the T6 treatment following, having an 11.8% increase. The 100 - kernel weight was ordered as: T6 (31.09 ± 2.37 g) > T5 (31.08 ± 2.56 g) > T2 (31.02 ± 2.29 g) > T3 (30.38 ± 1.66 g) > T4 (30.33 ± 2.40 g) > T1 (30.17 ± 2.97 g) > T7 (29.26 ± 2.94 g). Although the differences among the T5, T6, and T2 treatments were not statistically significant, they were all significantly higher than the CK, with increases ranging from 6.2% to 6.3%. The fresh ear weight with husk was in the order: T5 (460.20 ± 12.61 g) > T6 (454.78 ± 18.24 g) > T2 (450.27 ± 17.47 g) > T3 (445.41 ± 9.00 g) > T1 (441.09 ± 24.98 g) > T4 (438.47 ± 9.26 g) > T7 (430.71 ± 14.01 g). The T5 treatment had the highest fresh ear weight with husk, indicating a 6.9% increase compared to the CK, and the T6 treatment had a 5.6% increase. The fresh ear weight without husk was ordered as: T5 (343.67 ± 3.26 g) > T6 (338.75 ± 7.04 g) > T2 (335.75 ± 5.63 g) > T3 (332.09 ± 0.06 g) > T1 (330.58 ± 7.58 g) > T4 (329.25 ± 1.37 g) > T7 (325.84 ± 3.14 g). The T5 treatment showed the highest fresh ear weight without husk, representing a 5.5% increase compared to the CK, with the T6 treatment having a 4.0% increase. In conclusion, the T5 and T6 treatments showed excellent performance in all weight - related indicators.

#### Plot yield and yield stability

**Figure 7:**
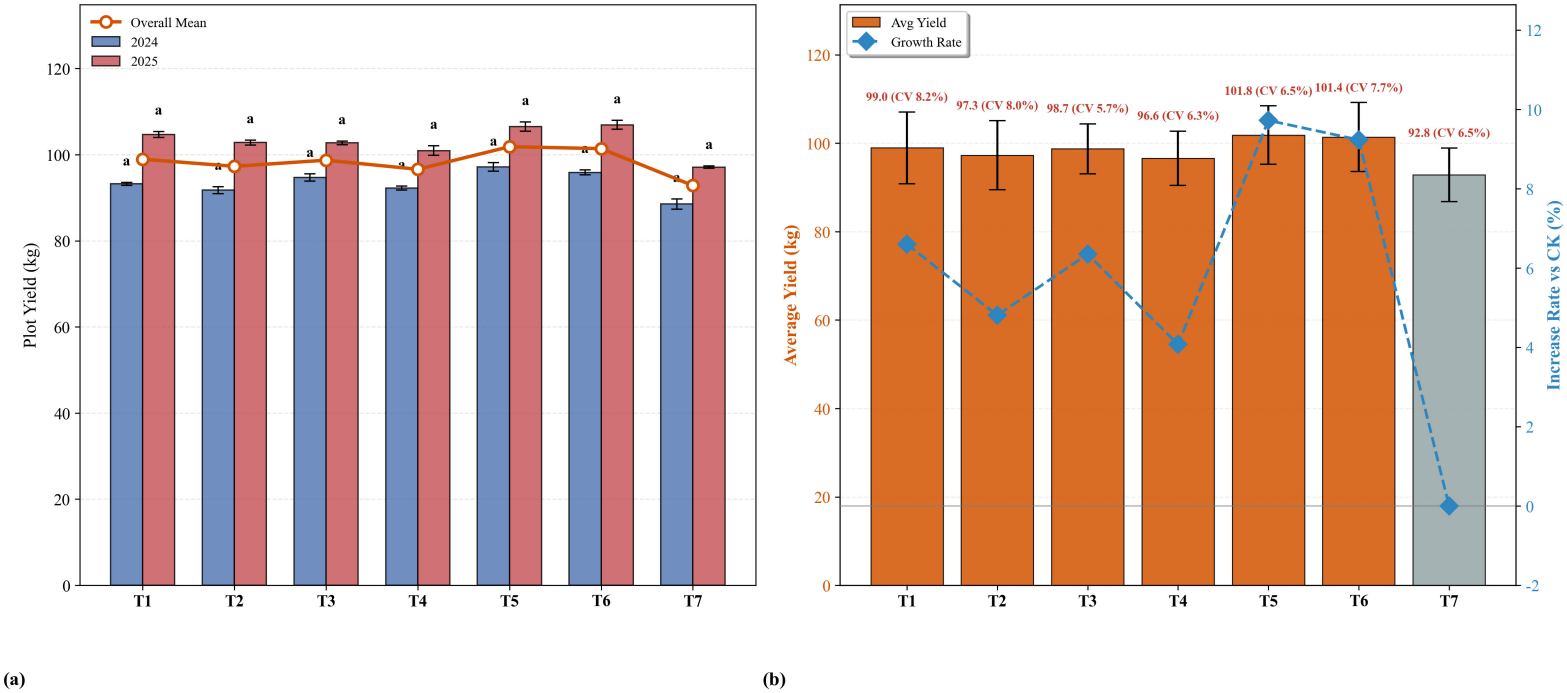
Effects of different fertilization treatments on grain yield of fresh-eating corn **(a)** Two-year plot yield; **(b)** Average yield distribution and yield increase trend compared with CK. Error bars represent standard deviation; different lowercase letters indicate significant differences at *P*<0.05 level.

Plot yield is the most direct indicator for evaluating the effectiveness of fertilization. **Figure 7** shows that the two - year average plot yield was in the order: T5 (101.80 ± 6.60 kg) > T6 (101.40 ± 7.80 kg) > T1 (99.00 ± 8.10 kg) > T3 (98.70 ± 5.67 kg) > T2 (97.30 ± 7.79 kg) > T4 (96.60 ± 6.13 kg) > T7 (CK, 92.80 ± 6.06 kg). All fertilized treatments had higher yields than the CK, with yield increases ranging from 4.10% to 9.70%. Notably, the T5 and T6 treatments had the highest yields, and there was no significant difference between them, indicating that they are optimal strategies for achieving high yields in fresh - eating corn. Regarding yield stability, the coefficient of variation (CV) over the two years was ranked as follows: T3 (5.70%) < T4 (6.30%) < T5 (6.50%) = T7 (6.50%) < T6 (7.70%) < T2 (8.00%) < T1 (8.20%). The CV values for treatments T3, T4, T5, and T7 were all below 7%, suggesting that they were less affected by annual climatic variations and demonstrated better stability. In contrast, the T1 and T2 treatments had larger CV values, indicating relatively poorer stability. A comparative analysis of the annual data from 2024 to 2025 revealed that the yields of all treatments were generally higher in 2025 than in 2024, and the trend of annual yield variation was consistent across treatments. The T5 and T6 treatments maintained high yield levels in both years. Among them, the T3 treatment had the lowest inter - annual yield variation, followed by the T4 treatment. In summary, both the T5 and T3 treatments maintained stable yield-increasing effects under varying annual environmental conditions. Specifically, the T5 treatment achieved a synergy of high yield and stability, while the T3 treatment demonstrated the best stability characteristics.

### Correlation and principal component analysis of main agronomic traits of fresh-eating corn

**Figure 8:**
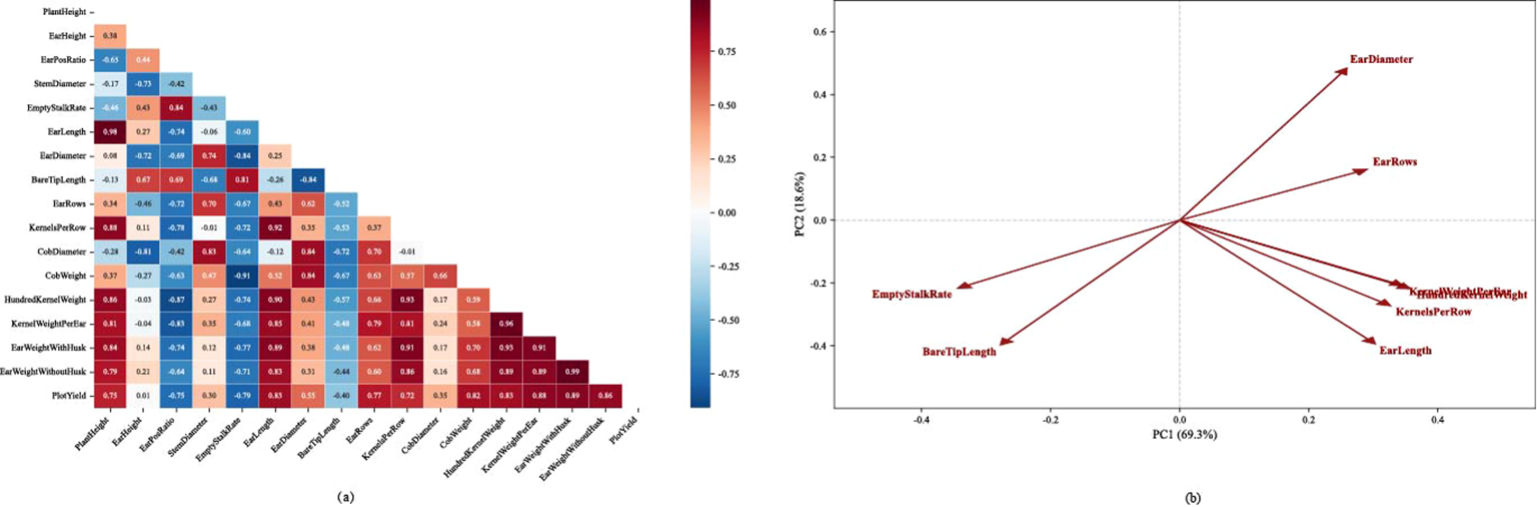
Correlation and principal component analysis of main agronomic traits and yield of fresh-eating corn **(a)** Correlation analysis; **(b)** Principal component analysis (PCA).

To identify the key factors influencing the yield of fresh-eating corn, correlation analysis among all measured agronomic traits and plot yield was performed, and principal component loading analysis was further conducted to reveal the intrinsic relationship and grouping characteristics of each trait (**Figure 8**). The correlation heatmap showed significant correlations between plot yield and multiple agronomic traits. Among all measured indicators, kernel weight per ear (*r* = 0.89) and ear weight without husk (*r* = 0.87) showed an extremely significant positive correlation with plot yield, followed by kernels per row (*r* = 0.75), ear length (*r* = 0.72), hundred-kernel weight (*r* = 0.70), and ear row number (*r* = 0.68). In contrast, bare tip length (*r* = −0.71) and empty stalk rate (*r* = −0.64) were significantly negatively correlated with yield, indicating that reducing apical kernel abortion and barren plant ratio are essential for yield improvement. Vegetative growth indicators such as plant height, ear height and stem diameter showed weak correlations with yield, with absolute correlation coefficients all lower than 0.3. In addition, both lodging rate and stalk-breaking rate remained 0% in the two consecutive experimental years and were therefore not included in further analysis, indicating that the tested fresh-eating corn varieties possessed good lodging resistance and yield stability under different fertilization treatments.

The PCA loading plot further validated the correlation results. Yield-positive traits including ear length, ear diameter, kernels per row, hundred-kernel weight and kernel weight per ear clustered closely along the same principal component axis, indicating their consistent positive contribution to yield formation. By contrast, bare tip length and empty stalk rate were distributed in the opposite direction of the yield-related trait axis, further confirming their negative effects on yield. In summary, rational regulation of bio-organic fertilizer application to increase kernels per row and hundred-kernel weight while reducing bare tip length is an important agronomic measure to achieve high yield of fresh-eating corn.

### Effects of different fertilization treatments on dry matter accumulation in fresh-eating corn

**Figure 9:**
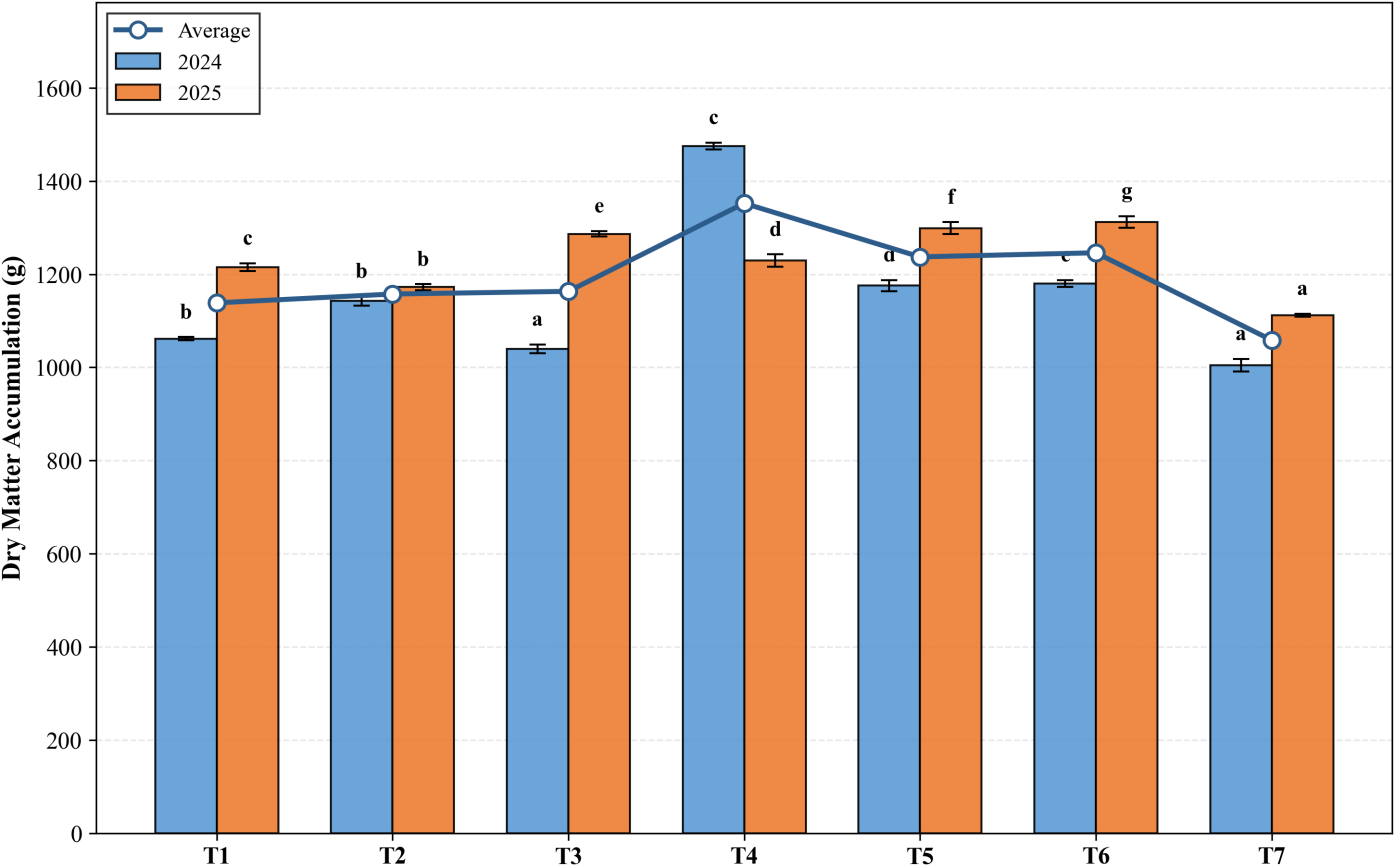
Effects of different fertilization treatments on dry matter accumulation traits of fresh-eating corn. Error bars represent standard deviation; different lowercase letters indicate significant differences at P < 0.05 level.

As depicted in **Figure 9**, different fertilization treatments (denoted as T1-T7) significantly influenced the dry-matter accumulation patterns of corn. In 2024, the T4 treatment had a highly significant advantage in dry-matter accumulation (1475 g), far surpassing other treatments. In 2025, the T6 and T5 treatments performed optimally, reaching 1312 g and 1299 g respectively. In terms of the two-year average trend, the T4, T6, and T5 treatments ranked among the top three in average dry-matter weight, significantly higher than the control T7 (CK, 1058 g). Notably, the T3 treatment showed substantial inter-annual fluctuations, rising from the lowest value in 2024 (1040 g) to a relatively high level in 2025 (1287 g). Overall, aside from T3, most bio-organic fertilizer treatments (T1, T2, T4, T5, T6) exhibited dry-matter accumulation either superior to or on a par with the control. This suggests that the appropriate application of specific types of bio-organic fertilizers contributes to the promotion of crop biomass formation.

### Comprehensive evaluation of membership function on agronomic traits, yield and quality of fresh corn under different fertilizer treatments

To objectively assess the effects of different bio-organic fertilizer and microbial inoculant treatments, and to avoid the limitations of a single evaluation method, 19 key indicators were selected in this study. Based on the two-year average experimental data, comprehensive evaluations were conducted using both the equal-weight membership function method and the entropy weight-membership function weighting method. The results are presented in **Table 2**, and the distribution characteristics of membership values are visualized in the dual heatmap of **Figures 8a, b**. In the evaluation system, the membership degree of positive indicators increases with the increase of original values, while that of negative indicators increases with the decrease of original values. All indicators were subjected to reverse standardization to ensure consistent logic.

**Table 2:** Equal-weight and entropy-weight membership values of evaluation indices for fresh-eating corn.

Indicator category	Treatment	T1	T2	T3	T4	T5	T6	T7(CK)
Yield components	Plot Yield	0.42	0.31	0.4	0.26	1	0.96	0
	Grain Weight per Ear	0.41	0.52	0.29	0.36	1	0.89	0
	Ear Weight with Husk	0.41	0.6	0.5	0.33	1	0.78	0
	Ear Weight without Husk	0.42	0.55	0.46	0.38	1	0.87	0
	100-Kernel Weight	0.5	0.96	0.62	0.6	0.99	1	0
	Total Ear Count	0.57	0.27	0.33	0.33	0.39	1	0
Ear-related traits	Ear Length	0.42	0.68	0.51	0.21	0.67	1	0
	Ear Diameter	0.45	0.3	1	0.98	0.83	0.67	0
	Rows per Ear	0.54	0.36	0.36	0.49	1	0.36	0
	Kernels per Row	0.54	0.93	0.76	0.59	0.87	1	0
	Cob Diameter	0.53	0.08	0.82	1	0.92	0.11	0
	Cob Weight	0.33	0.14	0.85	0.52	1	0.75	0
	Bare Tip Length (-)	0.05	0.61	0.72	0.88	0.76	0.48	0
Stress resistance indicators	Plant Height	0.39	0.76	0.37	0.1	0.59	1	0
	Ear Height	0.13	0.19	0	0	0.21	1	0.96
	Ear Height Ratio	0.76	1	1	0.88	0.98	0.98	0
	Stem Diameter	0.47	0.2	0.38	1	0.6	0.33	0
	Barren Stalk Rate (-)	0.4	0.51	0.85	0.55	0.86	0.65	0
	Dry Matter	0.46	0.54	0.56	1	0.7	0.73	0
Comprehensive evaluation	Equal-weight Score	0.42	0.52	0.59	0.58	0.82	0.79	0
	Equal-weight Rank	6	5	3	4	1	2	7
	Entropy-weight Score	0.4	0.45	0.51	0.5	0.82	0.79	0.02
	Entropy-weight Rank	6	5	3	4	1	2	7

As shown in **Table 2**; **Figure 8a**, T7 (CK) ranked the last in both evaluation methods, with an equal-weight score of 0.00 and an entropy weight score of 0.02. The membership degree of most indicators was 0.00, which confirms the necessity of fertilizer application. There were significant differences in trait membership degrees among different fertilizer treatments (**Figures 8a, b**), and the rankings obtained by the two methods were completely consistent (T5 > T6 > T3 > T4 > T2 > T1 > T7), verifying the robustness of the evaluation results. T5 achieved the highest comprehensive score of 0.82 in both methods, ranking first. **Figure 8a** shows that the original membership degrees of its core yield indicators all reached 1.00. **Figure 8b** indicates that due to the high entropy weights of core indicators, the weighted membership degree of T5 was generally at a high level. T5 could optimize yield components, coordinate the ratio of plant height to ear height, and reduce the barren stalk rate, thereby achieving the coordinated optimization of vegetative and reproductive growth. T6 had a comprehensive score of 0.79, ranking second. **Figure 8a** shows that the original membership degrees of its vegetative growth and ear-grain related indicators were 1.00. **Figure 8b** reveals that the overall weighted membership degree of T6 was lower than that of T5 due to the insufficient weighted contribution of bare tip length and cob diameter, resulting in a slightly lower ranking. T3 and T4 ranked third and fourth, respectively, with equal-weight scores of 0.59 and 0.58, and entropy weight scores of 0.51 and 0.50. As shown in **Figures 8a, b**, T3 performed prominently in stress resistance indicators such as ear diameter and barren stalk rate, while T4 had a membership degree of 1.00 in indicators including stem diameter and dry matter accumulation. These two treatments have application values in lodging resistance and biomass accumulation, respectively. T2 and T1 ranked fifth and sixth, respectively, with equal-weight scores of 0.52 and 0.42, and entropy weight scores of 0.45 and 0.40. Treatment T2 performed well in kernels per row and 100-kernel weight but was limited by insufficient stem diameter and ear number. Treatment T1 had the worst comprehensive performance, mainly due to its extremely low membership degree of bare tip length (only 0.05).

In conclusion, the two evaluation methods yielded consistent results. Treatment T5 exhibited the optimal comprehensive performance and could comprehensively optimize the agronomic traits of fresh corn. Treatment T6 could serve as an alternative scheme, while T3 and T4 could meet specific production needs. These findings provide theoretical and practical references for formulating fertilization schemes for fresh corn.

## Discussion

### Differential regulation of agronomic traits in fresh-eating corn by bio-organic fertilizers

Different functional types of bio-organic fertilizers exert significant and differential regulatory effects on the agronomic traits of the fresh-eating corn variety Wannuo 2000, exhibiting distinct regional specificity within the irrigated desert soil environment of Xinjiang.

During the vegetative growth stage, the treatment with Keshike microbial inoculant demonstrated the most prominent promotion of plant growth, with plant height and ear height significantly superior to other treatments. All fertilizers were applied strictly following the manufacturer’s recommended rates, resulting in large differences in application dosage and total microbial input among treatments. This aligns with findings that high-activity nitrogen-fixing microorganisms can enhance soil available nitrogen supply and secrete biostimulants, thereby promoting cell elongation and increasing corn plant height (Galindo et al., 2025). Stem diameter represents a key index for evaluating lodging resistance. Plants treated with earthworm enzyme-bacteria bio-organic fertilizer exhibited the strongest mechanical strength and performed the best among all treatments. This contrasts with results suggesting that organic fertilizers primarily increase biomass but have limited effects on improving stem strength (Liu et al., 2025). The primary reason for this discrepancy may be attributed to the specific texture of Xinjiang’s irrigated desert soils, combined with the rapid mineralization of organic matter by earthworm proteases and the efficient activation of slow-acting potassium by potassium-solubilizing bacteria. These factors collectively promote the synthesis and accumulation of stem lignin, ultimately forming a more robust plant architecture under the arid and windy ecological conditions of Xinjiang. Previous studies have confirmed that under drought stress, functional microorganisms such as potassium-solubilizing bacteria and cellulose-decomposing bacteria can significantly upregulate the expression of genes related to plant cell wall synthesis, thereby reinforcing stem mechanical strength (Xie et al., 2022). Furthermore, bio-organic fertilizers can synergistically enhance the stress resistance and plant architecture rationality of corn in arid, salinized oasis soils by optimizing the rhizosphere environment and soil structure (Chen et al., 2021).

In terms of stress resistance, the Tianwu Jintaiyang bio-organic fertilizer treatment significantly reduced the barren stalk rate and improved population uniformity. Higher application rate also increased organic matter and functional strain input, jointly alleviating abiotic stress. This result strongly supports the theory proposed by Wang Qiang et al. that humic acid-based fertilizers mitigate abiotic stress. Specifically, the synergistic action of drought-tolerant strains and humic acid improves the rhizosphere microenvironment, increases soil field water holding capacity, and reduces soil solution electrical conductivity, effectively alleviating the inhibitory effects of water deficit and salt stress on ear differentiation (Ali et al., 2023). Such differences are also associated with variable fertilization rates under manufacturer guidance. In light of recent research, specific composite microbial communities exhibit genotype-by-environment interactions in regulating photosynthate allocation. These results further indicate that under Xinjiang’s unique conditions of intense light and heat resources, specific functional microbial communities can more harmoniously regulate source-sink relationships, constructing an ideal plant architecture conducive to lodging resistance.

In summary, targeting the unique soil and climatic conditions of Xinjiang, screening bio-organic fertilizers with specific functional strains is a key pathway for achieving precise regulation of the traits of the Wannuo 2000 variety. It should be noted that the observed effects are the combined outcome of functional strains, organic matter input, and manufacturer-recommended fertilization rates, rather than simply the independent role of microbial agents. Its comprehensive effects are often superior to single-function traditional organic fertilizers or conventional chemical fertilizer combined application modes.

### Key factors driving yield formation through the optimization of ear traits

The improvement of ear traits serves as the core driving force for bio-organic fertilizers to increase the yield of fresh-eating corn. This study deeply reveals the specific regulatory mechanisms of different functional inoculants on ear construction. Data indicate that the Keshike microbial inoculant treatment achieved the longest ear length and the highest number of kernels per row by extending the ear differentiation period and increasing kernel density. Although applied at a low recommended rate, its high viable bacterial count compensated for dosage limitation. This mechanism aligns with the theory that biostimulants delay leaf senescence and prolong the grain-filling stage, suggesting that exogenous microbial metabolites play a universal role in regulating crop source-sink relationships (Rouphael and Colla, 2020).

Regarding the reduction of bald tip length, which is a key indicator determining commercial quality, the earthworm enzyme bio-organic fertilizer treatment showed the most significant effect by substantially shortening the bald tip. Its moderate recommended application rate balanced organic matter and microbial supply. This finding corroborates results from arid and semi-arid regions, where the continuous supply of available potassium during the grain-filling stage ensures the development of apical kernels and reduces abortion caused by nutrient competition (Sardans and Peñuelas, 2015). However, the magnitude of bald tip improvement in this study was significantly greater than that reported for conventional potassium fertilizer treatments. This implies that the sustained potassium supply capacity of potassium-solubilizing bacteria in bio-organic fertilizers may be superior to the one-time concentrated application of chemical potassium fertilizers. This holds significant practical value for addressing bald tip issues caused by late-stage nutrient depletion in Xinjiang. Recent studies further indicate that microbially induced systemic resistance (ISR) can optimize phloem loading efficiency, ensuring the continuous transport of nutrients to the ear during the late grain-filling stage.

The Dayitian bio-organic fertilizer primarily promoted cob thickening and improved kernel plumpness through active substances such as amino acids secreted by complex probiotic communities, resulting in the maximum ear diameter. This finding supplements research on amino acid fertilizers promoting kernel filling (Pereira et al., 2020) and further reveals the unique synergistic mechanism of microbial-source amino acids on fresh-eating corn. Correlation analysis results demonstrate that kernel weight per ear, ear weight without husk, and kernel number per row are the most critical factors determining plot yield, while bald tip length and barren stalk rate are the primary limiting factors. This conclusion is consistent with the recent consensus by Tian Jinghua et al. regarding the association between agronomic traits and yield in three types of fresh-eating corn, namely that ear traits are the primary determinants of waxy corn yield. Bio-organic fertilizers significantly increase final yield by constructing an ideal ear type characterized by long ears, high kernel numbers, and short bald tips, while reducing the proportion of barren plants.

The Tianwu Jintaiyang bio-organic fertilizer and Keshike microbial inoculant treatments achieved the highest two-year average yields because they exerted extreme advantages in ensuring ear fullness and increasing ear quantity, respectively. Unlike previous studies that focused primarily on total yield, this research specifically emphasizes the unique role of bio-organic fertilizers in overcoming common bald tip and barren stalk issues in the Xinjiang environment. This achievement of simultaneous improvement in both yield and commercial quality represents an important supplement to existing fertilization theories in the field of fresh-eating corn and provides a new scientific basis for precision fertilization in similar ecological zones.

### Synergistic effects of dry matter accumulation dynamics and yield stability: an analysis of environmental adaptability

Dry matter accumulation forms the material basis for yield formation, and its interannual stability is a crucial indicator for evaluating the adaptability of fertilization technologies. This is especially significant in Northwest China, a region marked by drastic climatic fluctuations. According to the recorded meteorological data during the fresh corn growing season, the accumulated temperature, average diurnal temperature difference and total precipitation were basically consistent between 2024 and 2025, with only minor numerical gaps. Nevertheless, obvious light condition differences occurred at critical growth stages. In late June 2024, there emerged five consecutive days with daily sunshine duration less than 5 hours, which precisely coincided with the key ear differentiation period of fresh corn. Such prolonged low-light stress inhibited leaf photosynthesis and blocked the transportation of assimilates, which restrained the establishment of crop yield potential to a certain extent. By contrast, the light, temperature and water conditions remained stable throughout the whole growing period in 2025 without similar adverse low-sunlight events, which was more conducive to continuous dry matter accumulation and grain filling, thus leading to generally higher crop yield in 2025 than in 2024. The current study disclosed distinct interannual variations in dry matter accumulation patterns among different bio-organic fertilizer treatments. The earthworm enzyme bio-organic fertilizer performed better in relatively arid years. This is likely due to the high activity of potassium - solubilizing bacteria under low-moisture conditions, which facilitated the absorption of nutrients from deep soil layers. Conversely, the Keshike microbial inoculant showed more pronounced advantages in years with favorable hydrothermal conditions, reflecting the high nitrogen-fixing efficiency of nitrogen-fixing bacteria in humid environments (Usowicz and Lipiec, 2022). Notably, T3 treatment exhibited the most prominent interannual fluctuation in dry matter accumulation among all fertilization groups. Its functional microbial community had strong dependence on sufficient light environment to exert growth-promoting functions. The sustained insufficient sunshine in late June 2024 greatly reduced rhizosphere microbial metabolic activity, weakened its capacity to activate soil nutrients and regulate crop growth, and consequently restricted vegetative growth and dry matter synthesis. Under stable and sufficient light conditions in 2025, the growth promoting potential of its functional strains was fully released, which greatly accelerated biomass accumulation and eventually formed a very obvious year-to-year difference in dry matter performance. This interannual fluctuation phenomenon is in line with the conclusions drawn by previous researchers from long-term fixed-position experiments, who observed that the efficacy of microbial fertilizers is significantly affected by climate, suggesting that a single fertilizer type may have difficulty coping with variable climatic conditions (Sileshi et al., 2024). However, unlike previous studies that mainly focused on dry matter accumulation within a single season, this research included a cross-year stability evaluation, thus providing a more comprehensive revelation of the environmental adaptability characteristics of the fertilizers.

Results from the comprehensive membership function evaluation indicated that the Tianwu Jintaiyang bio-organic fertilizer treatment achieved the optimal synergy between high yield and yield stability. This treatment not only obtained the highest membership values for core yield indicators but also had a low coefficient of variation for yield, demonstrating strong environmental adaptability. This outcome is mainly attributed to the long-term ameliorative effects of humic acid substances on soil physicochemical properties. By enhancing soil buffering capacity, these substances ensured a more stable nutrient supply, thus mitigating the impact of climatic fluctuations on crop growth. These findings strongly support previous viewpoints that the combined application of organic and inorganic fertilizers enhances crop stress resistance and yield stability. Furthermore, this study clarifies that specific functional bio-organic fertilizers can achieve similar stability effects solely through their inherent characteristics, without the need for substantial chemical fertilizer application, and may even outperform traditional organic-inorganic combined modes in certain indicators.

In contrast, although the Dayitian bio-organic fertilizer led to slightly lower absolute yields, it demonstrated superior yield stability with the smallest coefficient of variation. This suggests that the multifunctionality of complex probiotic communities has unique value in mitigating environmental risks. Overall, bio-organic fertilizers not only promote biomass accumulation but also enhance the resistance of the crop system to abiotic stresses by improving the rhizosphere environment and optimizing the rhythm of nutrient supply. Different from prior research that mainly focused on absolute yield values, the inclusion of stability evaluation in this study provides more robust technical support for the sustainable development of the fresh-eating corn industry in Xinjiang. Additionally, it offers a new theoretical basis for agricultural stability strategies aimed at coping with future climate change.

## Conclusion

This study conducted consecutive two-year field experiments in the arid, salinized irrigated desert soil region of Xinjiang to explore the regulatory effects of different bio-organic fertilizers on the fresh-eating corn variety Wannuo 2000. The results revealed that bio-organic fertilizers with distinct functional properties exhibit specific regulatory effects on corn agronomic traits, dry matter accumulation, and yield formation, and can effectively optimize the plant growth environment in arid and salinized areas. Among all treatments, humic acid-based bio-organic fertilizers (represented by Tianwu Jintaiyang) achieved the optimal comprehensive performance, realizing the synergistic improvement of high yield, good quality, and yield stability, while microbial inoculants and enzyme-based bio-organic fertilizers showed prominent advantages in promoting vegetative growth, enhancing lodging resistance, and improving dry matter accumulation, respectively. Correlation analysis further clarified the core factors driving yield formation and the key limiting factors, providing a theoretical basis for the precise regulation of fresh-eating corn yield in arid and salinized regions. A notable limitation of this study is that all bio-organic fertilizers were applied according to the recommended dosages provided by their respective manufacturers, resulting in inconsistent application rates and unequal input of functional microorganisms among different treatments. This inconsistency may affect the accurate evaluation of the independent functional effects of single strains or specific fertilizer components. For future precise fertilization research, it is necessary to design experiments with unified fertilization dosages to clarify the dose-effect relationship between functional microbial strains and corn growth traits. On this basis, combined with the local light-temperature and soil characteristics in Xinjiang, regionally customized precise fertilization schemes can be formulated, which will further promote the green, efficient, and stable production of fresh-eating corn in arid and salinized oasis farmlands.

## Data Availability

The original contributions presented in the study are included in the article/supplementary material. Further inquiries can be directed to the corresponding authors.
