# Correction: Effects of different bio-organic fertilizers on agronomic traits, yield, and dry matter accumulation of fresh-eating corn in arid and saline soils of Xinjiang: a single-site field experiment

**DOI:** 10.3389/fpls.2026.1902857

**Published:** 2026-07-09

**Authors:** Yanhua Cui, Yong Tang, Yao Du, Wanhong Ding, Hongsong Ren, Yulong Jia, Xuming Shi, Wei Wang, Shun Ji, Yibo Ma

**Affiliations:** 1 Xinjiang Uygur Autonomous Region Leisure Agriculture Engineering Research Center, Xinjiang Academy of Agricultural Sciences, Urumqi, China; 2 Urumqi Comprehensive Experiment Station, Xinjiang Academy of Agricultural Sciences, Urumqi, China; 3 College of Computer Science and Information Engineering, Anyang Institute of Technology, Anyang, China

**Keywords:** agronomic traits, bio-organic fertilizer, dry matter accumulation, fresh-eating corn, yield

Affiliation 1 “Xinjiang Uygur Autonomous Region Leisure Agriculture Engineering Research Center, Xinjiang Academy of Agricultural Sciences, Urumqi, China” was erroneously given as “Cotton Research Institute, Xinjiang Academy of Agricultural and Reclamation Science/Northwest Inland Region Key Laboratory of Cotton Biology and Genetic Breeding, Ministry of Agriculture and Rural Affairs, Urumqi, China”.

The original version of this article has been updated.

